# Uncertainty Effects on Smart Grid Services for Low-Voltage Distribution Networks

**DOI:** 10.3390/s26061800

**Published:** 2026-03-12

**Authors:** Federico Carere, Tommaso Bragatto, Alberto Geri, Silvia Sangiovanni, Marco Laracca

**Affiliations:** 1 Department of Information Science and Technology, Telematic University Pegaso, 80132 Naples, Italy; 2 Department of Electric and Energy Engineering, Sapienza University of Rome, 00184 Rome, Italy; tommaso.bragatto@uniroma1.it (T.B.); alberto.geri@uniroma1.it (A.G.); silvia.sangiovanni@uniroma1.it (S.S.); marco.laracca@uniroma1.it (M.L.)

**Keywords:** smart grids, voltage regulation, measurement uncertainty, sensor penetration, distributed energy resources, genetic algorithms

## Abstract

This study investigates the impact of monitoring infrastructure characteristics (specifically sensor penetration and measurement accuracy) on the effectiveness of voltage regulation and congestion management within distribution networks. As distribution system operators transition toward active management, the integration of Distributed renewable Generation (DG) and demand response introduces significant physical and cyber-physical uncertainties. To address these challenges, a smart grid service framework has been employed to optimize flexibility resources from aggregated users and DG inverters through a genetic algorithm. The framework was tested on the IEEE European Low Voltage Test Feeder across various scenarios defined by distributed monitoring systems’ penetration and their measurement accuracy. Results show that sensor penetration has a dominant impact: increasing monitoring coverage from 0% to 100% raises the percentage of cases with fewer than one residual congestion from 46.2% to 91.9% (sensors with an accuracy class of 2%), reaching 97.9% with an accuracy class of 0.5%, while voltage violations are eliminated under full monitoring. These findings suggest that widespread sensor deployment, with a suitable measurement accuracy, is a fundamental prerequisite for reliable and efficient smart grid operation.

## 1. Introduction

In recent years, distribution networks have undergone a profound transformation driven by the rapid increase in Distributed renewable Generation (DG), the electrification of end uses, and the widespread diffusion of electric vehicles [1,2]. These trends have significantly altered power flows and caused voltage variability along medium- and low-voltage feeders [3]. As a result, distribution system operators (DSOs) are shifting from traditional passive management to more active, data-driven control paradigms. Within this evolution, DG units equipped with modern inverters are no longer regarded solely as sources of renewable energy; instead, they are emerging as key resources for voltage regulation [4].

Voltage control at the distribution level traditionally relies on on-load tap changers, capacitor banks, and network reinforcement [5]. However, these solutions are slow, costly, and insufficient for managing fast variations induced by photovoltaic (PV) and wind generation. Consequently, leveraging the reactive power capability of DG units has become a central strategy. This can be done through local schemes, such as power factor control or voltage-dependent reactive power control, which allow inverters to modulate reactive power autonomously based on local measurements [6,7]. Such methods aim to mitigate overvoltages caused by high renewable injections, helping maintain voltage within regulatory limits without requiring additional grid infrastructure.

Although local control schemes are attractive for their simplicity and independence from communication systems, their impact on the overall network is often limited. Several studies suggest that in environments characterized by a high penetration of DG (high-DG), coordinated or centralized voltage regulation strategies can achieve better results by exploiting global grid information and adjusting reactive power dispatch accordingly [8]. Additionally, time-dependent reactive power setpoints have been proposed to compensate for reactive power flows at primary substations and reduce related costs imposed by national regulators [9,10]. These approaches show promising potential but require accurate knowledge of loading conditions to avoid unintended voltage violations along feeders.

Beyond voltage regulation, flexibility in consumption and generation has emerged as an additional tool to support network operation. Demand response (DR) programs allow users to temporarily modify their consumption to alleviate congestion, reduce peak loading, and resolve voltage issues [11,12]. However, the effectiveness of flexibility-based solutions depends heavily on the uncertainty associated with user behavior. Even when flexibility is procured through local markets or aggregators, users may not provide the expected response due to behavioral factors, limited controllability, lack of appliances, or communication issues. This uncertainty has been widely recognized as a major barrier to large-scale DR adoption [13].

Uncertainty affects voltage control through several channels. First, fluctuating loads and renewable production directly influence voltage magnitude, potentially invalidating reactive power setpoints calculated on expected conditions. Second, flexibility that is promised but not delivered may leave the system unprotected against congestion or voltage deviations. Third, when DG inverters are expected to provide reactive support, their actual capability depends on instantaneous active power production, which is itself uncertainty for PV and wind units. These uncertainties make voltage regulation a probabilistic challenge rather than a deterministic one, pushing researchers toward robust or stochastic control approaches [14,15].

In addition, voltage regulation strategies increasingly rely on real-time measurements collected from smart meters, field sensors, and inverter controllers. However, in practical distribution networks, these data streams are affected by uncertainty because of several reasons such as measurement noise, calibration errors, communication delays, packet losses, and missing values, which are common issues that can significantly affect the reliability of the control process. Since voltage regulation algorithms compute reactive power setpoints directly from these inputs, even limited inaccuracies may propagate through the control chain and lead to suboptimal or unstable voltage profiles.

Beyond these inherent measurement uncertainties, the situation can be further worsened by intentional data manipulation which exploits the same communication and sensing infrastructure. In this regard, reference [16] highlights how centralized voltage regulation schemes can be particularly sensitive to corrupted measurement data. By injecting false demand or generation values into the state estimation process of a high-DG network, the authors show that even moderate distortions in the measurement set can cause the controller to compute incorrect reactive power commands. This may result in significant voltage deviations, inefficient inverter operation, and, in extreme cases, service disruptions at secondary substations.

Their findings also indicate that vulnerability increases with higher DG penetration, since voltage control becomes progressively more dependent on inverter-based resources whose response is tightly coupled with the accuracy and timeliness of network measurements. Overall, this emphasizes that DSOs must address not only the variability of loads and renewable generation but also the broader uncertainty affecting measurement and communication systems. Improving data quality through redundancy, validation mechanisms, anomaly detection, and secure communication protocols therefore becomes essential to ensure robust and trustworthy voltage regulation in active distribution networks.

Another key trend of distribution network operation is the development of a Local Flexibility Market. The transition is necessitating a shift for DSOs, moving them from traditional “fit and forget” network expansion toward the active management of their grids through market-based mechanisms [17]. Within this evolving landscape, Local Flexibility Markets (LFMs) have become a tool for procuring ancillary services from Distributed Energy Resources, with congestion management consistently identified as the most prevalent and critical service across European pilot projects. These services primarily aim to resolve thermal overloads on feeders and voltage violations by incentivizing prosumers and aggregators to temporarily adjust their consumption or production patterns [18].

Current LFM initiatives across Europe vary significantly in maturity, with Great Britain and the Netherlands currently hosting the most advanced operational schemes. In the UK, all six DSOs utilize platforms like Piclo Flex [19] and Electron Connect [20] to procure standardized active power services such as Peak Reduction and Scheduled Utilization. The Dutch GOPACS [21] platform provides a distinctive intermediary model that coordinates congestion management between TSOs and DSOs. More recent developments in Southern Europe include Italy’s RomeFlex and MiNDFlex pilots [22,23], alongside Portugal’s FIRMe project [24], all of which focus on localized active power regulation.

Despite the great interest of operators and researchers in smart grid services for voltage regulation and congestion reduction, some topics are still unexplored. On the one hand, the effects of uncertainty on the performances of the smart grid services are often neglected and not systematically assessed. On the other hand, limited attention has been paid to the application of these smart grid services on low-voltage (LV) distribution networks, even though these networks can suffer from congestions and their monitoring systems are under development.

A limited number of papers are focused on these topics, as in [25,26]. Although these papers have already underlined the impact of uncertainty sources on smart grid services, the impact of monitoring uncertainty on combined smart grid services for LV grids has not been investigated yet. Other works analyze additional uncertainty sources related to flexibility exploitation. As an example, in [27], the impact of the probabilistic characteristics of uncertainty variables on flexibility evaluation is assessed; in [28], the impact of customer responsiveness on network congestions is evaluated. However, a recent review [29] reports the need to develop uncertainty modeling methods suitable for novel tasks to tackle emerging issues, such as those introduced by smart grid services.

This work proposes the assessment of uncertainty effects on a smart grid service framework specifically designed for voltage regulation and congestion management tasks. This framework is conceived to identify and optimize the allocation of available flexibility resources, deriving from both user load profiles and the capabilities of distributed generation inverters, with the explicit objective of eliminating voltage violations and resolving network line congestions. The system is tested under various forms of uncertainty, focusing particularly on the impact of the monitoring infrastructure in terms of sensor penetration and measurement accuracy. By applying the framework to the IEEE European LV Test Feeder [30], this study provides insights into the robustness of the control strategies required to ensure secure grid operation even in the presence of partial visibility.

The remainder of this paper is organized as follows: Section 2 describes the characteristics of modern monitoring systems for distribution networks and the relevant accuracy classes; Section 3 presents the architecture of the smart grid service framework and the optimization algorithm used for resource management; Section 4 illustrates the evaluated scenarios based on the penetration and accuracy of the distributed monitoring systems; Section 5 describes the case study based on the IEEE benchmark; Section 6 presents and discusses the results obtained in terms of residual congestions, voltage profiles, and required flexibility power; and finally, Section 7 draws the conclusions of the work.

## 2. Monitoring Systems for LV Distribution Networks

LV grids are currently facing significant renovation of assets and equipment for their monitoring and control. As an example, in [3], it is reported that 50% of the installed PV capacity in distribution networks is connected to LV grids. According to [3,31], key trends for the monitoring infrastructure development of LV distribution networks are: (i) the deployment of Advanced Metering Infrastructures (AMIs); (ii) the installation of innovative sensors for low-cost network monitoring; (iii) the development of forecasting algorithms for increasing network observability and controllability. In this paper, the above-mentioned key trends were respectively analyzed considering: (i) the share SH, consisting of the percentage of the grid nodes that are monitored by distributed measurement systems; (ii) the accuracy ACC, representing the accuracy in the power measurement of the low-cost network monitoring systems; the predictive measurements PMs, which are an estimation of the power measurements in the grid nodes characterized by the absence of the monitoring systems.

AMIs are the backbone of the smart grid and allow for two-way communication between smart meters and utility companies [32]. According to [3,33], smart metering deployment has reached an average coverage of 74% across the EU, representing approximately 134 million supply points. Currently, AMIs are no longer merely billing tools but are used for grid visibility, enabling additional services. As an example, 58% of the DSOs surveyed in [3] are leveraging AMIs for network state analysis and topology optimization, while 29% have integrated them into state estimation processes. Reference [31] reports the most promising use cases that could exploit AMI are: the support for LV network operation (e.g., improvement in voltage profile, remote management of loads, balancing of consumption and production, and fault detection and location); network efficiency improvement; quality of service (e.g., power quality, reliability, and minimization of power interruptions); management cost improvement (e.g., OPEX reduction and investment planning) and regulatory obligations.

Despite significant progress, full coverage across the LV distribution grid remains limited. References [3,34] indicates that only 20–40% of MV/LV substations in urban networks are remotely controlled, while the proportion falls to 18–25% for large DSOs serving more than 10 million customers. Therefore, in order to increase network observability, several devices are under development for increasing the number of monitored lines and nodes. As an example, several European DSOs are exploring the use of electronic LV switchgear installed in the LV compartment of secondary substations to enhance field observability. These systems provide real-time LV feeder measurements and comply with IEC 61557-12, which requires an accuracy class of 2% for power and energy measurements [35,36]. In addition, low-cost monitoring devices are under investigation to support smart grid services, as in [37,38,39,40].

To compensate for this incomplete observability, voltage regulation strategies often incorporate PM based on historical demand profiles, forecasted generation patterns, and statistical reconstructions of unmonitored nodes. PMs are widely used to approximate real-time conditions in areas where physical sensors are not yet installed. However, as documented in [39], the accuracy that can be associated with such predictive inputs is significantly lower than that of physical instrumentation, often around 50% under practical operating conditions. For this reason, PM-based information is modeled as the lowest-quality data source in the voltage regulation framework. It is worth mentioning that these accuracies do not consider the effect of FDIA since the aim of this work is to assess only the impact of power measurement uncertainties.

The limited penetration of measurement infrastructure implies that smart grid services frequently operate under incomplete or sparse visibility, increasing the dependence on less accurate data sources. On the one hand, voltage regulation systems may rely on a second tier of monitoring data with medium accuracy (2%); on the other hand, the pseudo measurements introduce considerable uncertainty even though they remain essential during the transition phase in which DSOs are progressively expanding their monitoring infrastructure. This heterogeneity directly affects the precision of control actions, the reliability of reactive power dispatch commands, and the capability of the system to maintain voltage stability in the presence of rapid fluctuations in load and renewable generation.

## 3. Smart Grid Service Framework

To enable the utilization of flexibility resources for the operation of distribution networks, the authors developed a software tool—referred to as a smart grid service framework—aimed at mitigating congestion in LV networks. The development of the smart grid service framework adheres to standardized flexibility platform architectures and data models and is based on the following assumptions:

(i) The tool is applicable exclusively to LV distribution networks;
(ii) Active and reactive power measurements are available at LV consumption/production nodes;
(iii) All LV active and passive users provide a quantifiable degree of flexibility, allowing aggregated users to modulate their power consumption within predefined boundaries;
(iv) Flexibility activation is implemented through predefined percentage-based variations in consumption;
(v) The cost associated with flexibility activation is computed exclusively based on the contributions of the LV loads;
(vi) Network reconfiguration is excluded as a control action, under the assumption that the LV network has no reconfiguration capability.

**Figure 1:**
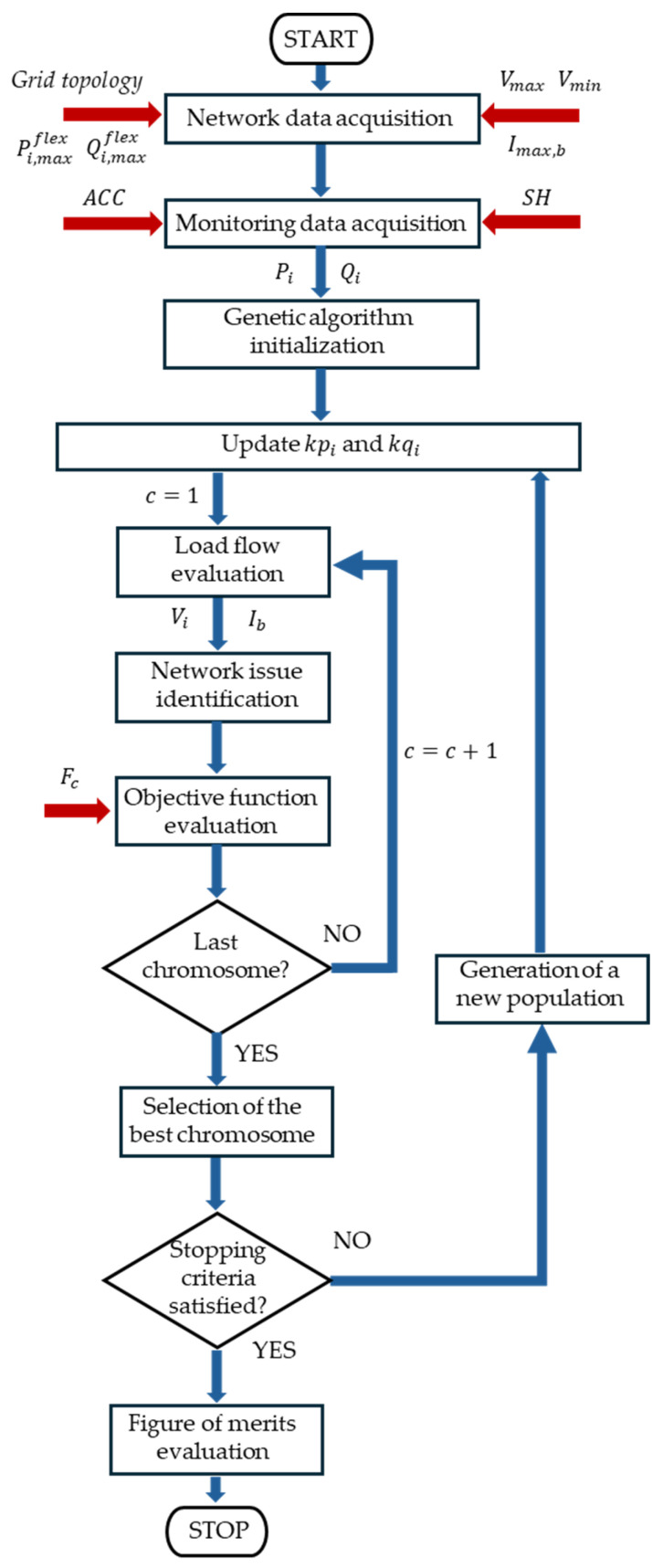
Flowchart of the smart grid service framework for the optimal allocation of flexibility resources; the red arrows highlight the main input of the framework.

**Figure 2:**
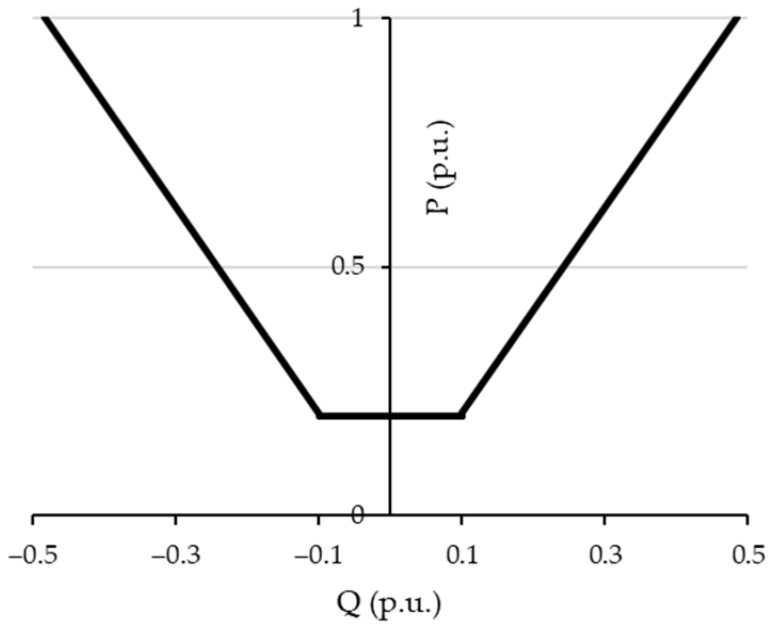
Capability curve assumed for DG in the smart grid service context.

The smart grid service framework was implemented in a MATLAB R2025b environment following the flowchart illustrated in Figure 1. In the beginning, the network data are acquired as an input of the procedure. They consist of the topology of the distribution grid, the power availability for the flexibility service and the technical constraints related to the voltage and current limits. With respect to the power availability for the flexibility service, the procedure considers the fact that flexible users can be load consumers that can reduce the power absorbed by a photovoltaic power plant and can exploit the capability of the inverters as reported in their related capability curves (as shown in Figure 2). For this reason, the smart grid service framework considers the maximum active ($P_{i,\max}^{\mathrm{flex}}$) and reactive ($Q_{i,\max}^{\mathrm{flex}}$) powers available at each node i of the network for the flexibility service. With respect to the technical constraints, the maximum voltage $V_{\max}$, the minimum voltage $V_{\min}$ and the ampacity $I_{\max,b}$ of each branch b of the network are provided for the procedure in order to identify the network issues.

The network issues can be a branch overload or a voltage violation. Notably, a voltage violation occurs in node i of the distribution grid if the following condition is not fulfilled:

V_min_ < V_i_ < V_max_ (1)

where V_i_ represents the voltage magnitude at node i of the network and V_min_ and V_max_ are the minimum and maximum allowable values of the voltage magnitude (that can be assumed to be 0.95 pu and 1.05 pu respectively).

As far as the branch overload is concerned, notably, branch b of the distribution grid is not considered overloaded if it fulfills this condition:

I_b_ < I_max,b_ (2)

where I_b_ represents the current magnitude that flows through line b of the network.

After acquiring the network data, the smart grid service framework needs the distributed power measurements at each node i of the distribution grid (i.e., the active power P_i_ and the reactive power Q_i_). In order to simulate the load conditions of the grid, the procedure performs a random generation of power measurements, starting from the knowledge of the accuracy ACC of the distributed monitoring systems of the grid and the share (SH). The SH consists of the percentage of nodes that are monitored by measurement systems characterized by an accuracy ACC. The remaining nodes are assumed to be unmonitored (i.e., nodes characterized by PM with an accuracy of 50%). The generation of the active power P_i_ and the reactive power Q_i_ is performed by means of a random extraction among the uncertainty range associated with power measurements. According to the monitoring system analysis reported in Section 2, the power measurement quality of the distributed monitoring systems is defined in terms of accuracy. In the absence of other information about the distribution power measurement quality, a common approach is applied assuming a uniform probability distribution that allows us to evaluate the standard uncertainties of the power measurements as $\mathrm{ACC}/\sqrt{3}$.

The smart grid service framework exploits the acquired network and monitoring data in order to minimize the resources that solve all the network issues (i.e., branch overloads and voltage violations). This target requires us to solve an optimization problem characterized by non-linear constraints, due to the need to do the load flow calculations, and by relevant computational effort. In this context, genetic algorithms (GAs) have demonstrated superior performance compared to stochastic techniques like the Monte Carlo approach, as they are specifically engineered for global optimization and exhibit high computational efficiency in tackling complex, real-world problems [40]. In this paper, a GA is implemented considering a chromosome consisting of a vector merging the variables kp_j_ and kq_j_ that is to be optimized. The variable kp_i_ is the adopted fraction of the maximum active power $P_{i,\max}^{\mathrm{flex}}$ available at node i of the network for the flexibility service, whilst kq_i_ is the adopted fraction of the maximum reactive power $\;Q_{i,\max}^{\mathrm{flex}}$. To achieve the aim of this research, a discretization of 10% of the flexibility power request is defined. The adopted GA is characterized by a population size of 30, a stochastic uniform selection, and a crossover probability of 0.8 and a mutation probability of 0.2. After randomly generating the initial population, the GA starts an iterative process in which the objective function F_c_ is evaluated for each chromosome c. In this iterative process, the algorithm carries out the load flow of the distribution grid using the OpenDSS tool, providing the voltage Vi at each node i and the current I_b_ of each branch b of the grid. The voltage and current evaluations are used to identify the network issues according to inequalities (1) and (2). After network issue identification, the GA is capable of evaluating the objective function F_c_ to be minimized, which is formulated as follows:

(3) $$F_{c}\;=\;F_{\mathrm{bin}}\cdot C+\sum_{i}^{N}({\mathrm{kp}}_{i}\cdot P_{i,\max}^{\mathrm{flex}}+{\;\mathrm{kq}}_{i}\cdot Q_{i,\max}^{\mathrm{flex}})$$

In Equation (3), $F_{\mathrm{bin}}$ represents a binary value which is 0 or 1 if the network issues are still absent or present, respectively; C represents an arbitrary quantity, expressed in kVA, that is high enough to represent the fact that the fulfillment of the network constraint is more important than minimizing the resources (i.e., a value equal to the overall available flexibility power); and N is the number of nodes in the grid. The optimization module is subsequently developed to determine the most efficient allocation of flexibility resources, with the objective of resolving the highest possible number of network issues. If inequality (1) is not fulfilled by even one of the branches, $F_{\mathrm{bin}}$ is counted as 1. If inequality (2) is not fulfilled for all the nodes of the network, $F_{\mathrm{bin}}$ is counted as 1. It can be stated that inequalities (1) and (2) represent implicit constraints to the optimization problem. At the end of the iterative process referred to as the objective function evaluation, the GA selects the best chromosome and verifies if the stopping criteria are met. In this paper, the stopping criteria of the function tolerance, defined as an upper bound on the step-to-step variation in the objective function value, and of constraint tolerance, defined as an upper bound on the magnitude of any constraint violation, are implemented within fixed threshold values, respectively, of 10^−6^ and 10^−3^. If both the stopping criteria are not satisfied, the GA generates a new population, updates the variables kp_i_ and kq_i_ and restarts the iterative process of the objective function evaluation. Once any stopping criteria are satisfied, the best chromosome is retained and reported as the solution, which corresponds to the optimal flexibility allocation. The solution is exploited to evaluate the figures of merit of the smart grid service framework, which consist of the number of residual congestions, the number of voltage violations and the adopted flexibility power.

## 4. Evaluated Scenarios

The smart grid service framework illustrated in Section 3 is adopted to assess, in different scenarios, the uncertainty impact on the smart grid services. Scenarios are defined as sets of 100,000 simulations designed to investigate the impact of monitoring infrastructure characteristics on the performance of the state estimation process. In particular, the proposed scenarios aim at assessing how both the penetration level of measurement devices and their accuracy affect the quality of the estimated network states. The simulated scenarios are fully described in terms of share SH and measurement accuracy ACC, as summarized in Table 1.

Scenarios with an SH equal to 0% represent limiting conditions in which no measurement devices are available in the network. In these cases, the state estimation relies exclusively on non-instrumented information. As the value of the SH increases, a progressively larger portion of the network is directly monitored by sensors with the specified accuracy, leading to an increasing level of observability. The extreme case of an SH equal to 100% corresponds to full deployment of sensors across all network nodes, resulting in complete measurement coverage at the given accuracy level.

For all scenarios with an SH greater than 0%, the placement of measurement devices is not performed randomly. Instead, sensors are optimally allocated within the network in order to maximize the accuracy and robustness of the state estimation results. The optimal placement is determined using the algorithm proposed in [41], which aims at selecting the most informative nodes from the perspective of state observability and estimation precision. Once the optimal locations are identified, the same accuracy level ACC is assigned to all sensors deployed in the corresponding scenario, in accordance with the values reported in Table 1.

It is worth emphasizing that no uncertainty is assumed for the network model parameters in any of the simulated scenarios. Line impedances, network topology, and all other electrical parameters are considered perfectly known. This modeling choice allows us to isolate the effects of measurement-related uncertainty and sensor penetration, ensuring that the observed variations in state estimation performance can be directly attributed to changes in SH and ACC, without interference from additional sources of uncertainty.

Overall, the adopted scenario definition enables a systematic and controlled analysis of the trade-off between measurement accuracy and monitoring coverage, providing insights into the relative importance of sensor quality and sensor deployment density in distribution network state estimation.

## 5. Case Study

The whole procedure is applied to the IEEE European LV Test Feeder. This standard model is explicitly implemented within the Open Distribution System Simulator (OpenDSS) environment, leveraging its capabilities for unbalanced three-phase power flow analysis.

**Figure 3:**
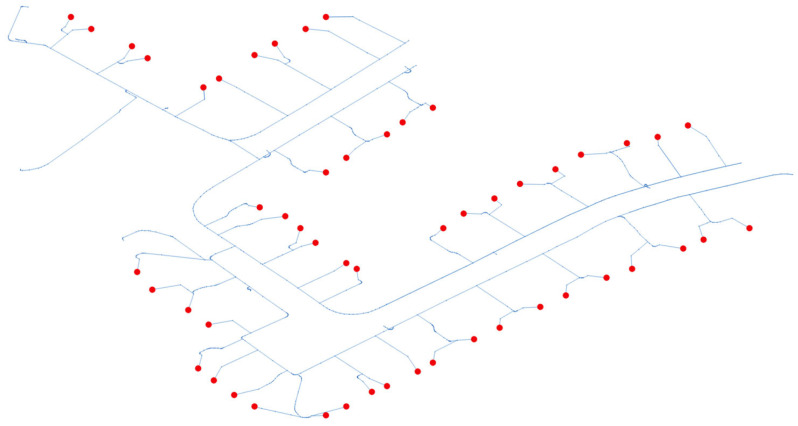
Schematic representation of the IEEE European LV Test Feeder used for the case study. Red dots represent the LV loads.

Figure 3 illustrates the schematic layout of the IEEE European LV Test Feeder utilized for the case study. The feeder reflects the fundamental characteristics of typical European LV distribution networks. It operates at a nominal voltage of 400 V (line-to-line)/230 V (line-to-neutral) with a 50 Hz nominal frequency. The model is typically supplied from a medium-voltage network through an MV/LV transformer configured in a Delta-Wye connection, which provides the essential grounded neutral point.

In particular, the system employs a four-wire configuration (three phases plus the neutral conductor). Since most of the connected loads (residential and small commercial) are modeled as 230 V single-phase connections between a phase and the neutral, the explicit modeling of the neutral conductor allows for the evaluation of load unbalances, neutral current and voltage quality.

The IEEE European LV Test Feeder serves as a standardized benchmark for assessing the integration and impact of modern grid challenges. Its use is particularly relevant for studying high-penetration scenarios of DERs and the increasing adoption of electric vehicles (EVs). By using this established model, the research ensures that the results are repeatable and comparable with other studies addressing power quality, voltage regulation, and network losses in real-world European smart grid contexts.

## 6. Results

**Figure 4:**
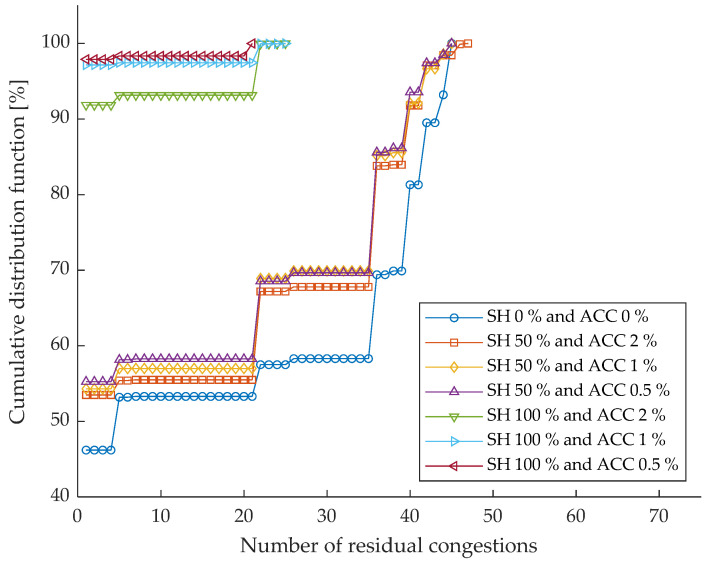
Cumulative distribution function of the number of residual congestions across different monitoring scenarios (SH and ACC).

Considering the network described in Section 5, the test conditions defined in Table 1 are used to perform several simulations using the optimizer presented in Section 3. The results of this analysis, both in terms of residual congestion and voltage violations, are shown below. In particular, Figure 4 reports the cumulative distribution function (CDF) of the number of residual congestions obtained under the different monitoring scenarios defined by their share and measurement accuracy. Each curve represents the statistical distribution of residual congestions over the simulated cases, allowing for a direct comparison of the effectiveness of different monitoring configurations. The corresponding quantitative results are summarized in Table 2, which reports the percentage of cases in which the number of residual congestions is lower than one.

In the case of the benchmark scenario (i.e., ideal monitoring system characterized by null uncertainty), the number of residual congestions is equal to zero, confirming that the available flexibility power is sufficient to solve the congestions. The worst scenario, corresponding to an SH equal to 0% and ACC equal to 0%, represents the absence of monitoring devices in the network. In this case, the cumulative distribution function is clearly shifted towards higher values of residual congestions, indicating the limited capability of the state estimation process to correctly identify and mitigate congestion conditions. This behavior is confirmed by the numerical results in Table 2, where only 46.2% of the simulated cases result in fewer than one residual congestion.

Introducing monitoring devices on 50% of the network nodes (SH = 50%) already produces a noticeable improvement in performance, regardless of the adopted accuracy level. As shown in Figure 4, all SH = 50% curves are shifted leftwards with respect to the worst scenario (i.e., SH = 0%; ACC = 0%) indicating a reduction in the number of residual congestions. The table confirms this trend, with the percentage of successful cases increasing to 53.5%, 54.3%, and 55.3% for ACC values equal to 2%, 1%, and 0.5%, respectively. Although the differences among the three accuracy levels are relatively limited at this penetration level, a slight performance gain can be observed when higher-accuracy measurements are employed.

A much more pronounced improvement is observed when the monitoring penetration reaches 100%. In this case, the CDFs exhibit a steep increase at very low values of residual congestions, demonstrating that full observability of the network significantly enhances the effectiveness of the state estimation process. According to Table 2, the percentage of cases with fewer than one residual congestion rises to 91.9% for ACC equal to 2% and exceeds 97% for ACCs equal to 1% and 0.5%. This result highlights how a high level of sensor penetration strongly mitigates congestion persistence, even when measurement is not extremely accurate.

Comparing scenarios with the same SH but different ACC values, it emerges that measurement accuracy has a secondary, yet non-negligible, impact on performance. While increasing accuracy consistently improves the results, its effect becomes particularly evident at high penetration monitoring levels, where the combination of full observability and accurate measurements leads to near-optimal congestion mitigation.

Overall, the figure and the table jointly demonstrate that sensor penetration plays a dominant role in reducing residual congestions, while measurement accuracy acts as a performance enhancer. These results suggest that, from a planning perspective, ensuring sufficient coverage of monitored nodes is a key prerequisite for effective congestion management, whereas improvements in sensor accuracy provide additional benefits once a high level of observability has been achieved.

**Figure 5:**
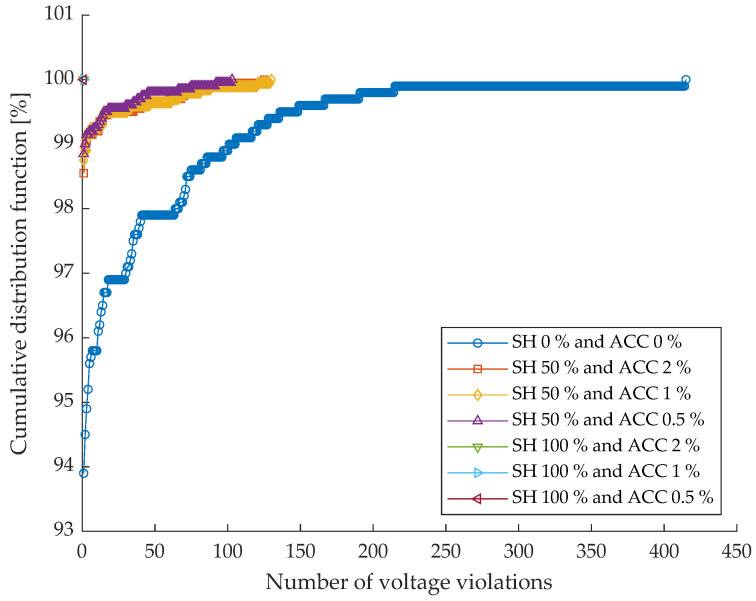
Cumulative distribution function of the number of voltage violations across different monitoring scenarios (SH and ACC).

Figure 5 shows the cumulative distribution function of the number of voltage violations obtained under the different monitoring scenarios characterized by their share and measurement accuracy. Each curve represents the statistical distribution of voltage violations over the simulated cases, while Table 3 reports the percentage of cases in which the number of voltage violations is lower than one.

In the case of the benchmark scenario (i.e., ideal monitoring system characterized by null uncertainty), the number of voltage deviations is equal to zero, confirming that the available flexibility power is sufficient to solve the voltage issues. The worst scenario (i.e., SH = 0%; ACC = 0%), corresponding to the absence of monitoring devices, exhibits the poorest performance. Although most of the cases still present a limited number of voltage violations, the CDF is noticeably more spread along the horizontal axis compared to the monitored scenarios, indicating the occurrence of a non-negligible number of cases with multiple voltage violations. This behavior is reflected in Table 3, where only 93.9% of the cases satisfy the condition of fewer than one voltage violation.

Introducing monitoring devices on 50% of the network nodes leads to a significant improvement in voltage regulation performance. All scenarios with SH = 50% show a sharp rise in the CDF at very low values of voltage violations, indicating that most of the simulated cases are characterized by no residual violations. The numerical results confirm this trend, with the percentage of successful cases increasing to 98.6%, 98.8%, and 98.9% for ACCs equal to 2%, 1%, and 0.5%, respectively. As observed in Figure 5, the impact of measurement accuracy at this monitoring level is relatively limited, with only marginal differences among the curves.

When full monitoring coverage is considered (SH = 100%), the performance further improves and becomes almost insensitive to measurement accuracy. Indeed, all SH = 100% curves overlap and reach a cumulative probability of 100% at zero voltage violations. This result is confirmed by Table 3, where all scenarios with SH = 100% achieve 100% of cases with fewer than one voltage violation, regardless of the adopted ACC value. This indicates that full observability of the network is sufficient to completely eliminate residual voltage violations in the considered test cases.

Overall, the results show that voltage violations are strongly mitigated even at moderate levels of sensor penetration and that increasing the SH has a dominant effect with respect to improving measurement accuracy.

By jointly analyzing the results on residual congestions and voltage violations, a clear difference emerges in the sensitivity of the two performance indicators to monitoring infrastructure characteristics. In both cases, increasing the share consistently improves system performance, confirming the fundamental role of sensor penetration in enhancing observability and control effectiveness.

However, voltage violations appear to be less critical than residual congestions in the worst scenario and are more easily mitigated through partial monitoring. Even with an SH equal to 50%, most of the cases already exhibit no voltage violations, and full observability guarantees their complete elimination independently of measurement accuracy. In contrast, residual congestions show a stronger dependence on both SH and ACC, with a substantial performance gap between partial and full monitoring scenarios and a more pronounced benefit associated with higher measurement accuracy at high SH levels.

These results suggest that voltage regulation is more robust with respect to limited observability, while congestion management requires a denser and more accurate monitoring infrastructure to achieve comparable performance. From a system planning perspective, this implies that moderate sensor deployment may be sufficient to ensure acceptable voltage profiles, whereas a more extensive and accurate monitoring infrastructure is necessary to effectively address congestion-related issues.

**Figure 6:**
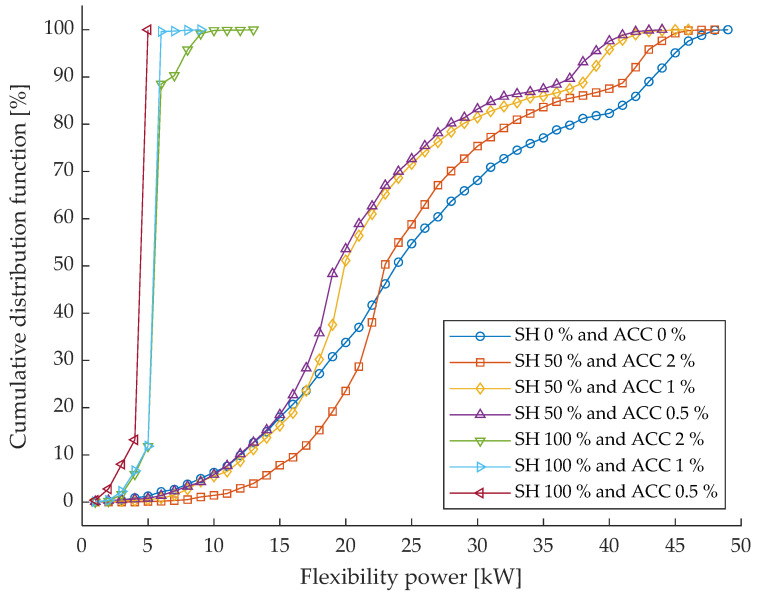
Cumulative distribution function of the flexibility power required to solve network constraints under various monitoring scenarios.

Figure 6 reports the cumulative distribution function of the flexibility power required to solve network constraints under the different monitoring scenarios defined by their share and measurement accuracy. The flexibility represents the amount of control action activated to ensure secure network operation. Table 4 summarizes the percentage of cases in which the activated flexibility power is lower than the reference value evaluated for the benchmark scenario (i.e., ideal monitoring system characterized by null uncertainty), which corresponds to a power of 45.83 kW.

The worst scenario (SH = 0%, ACC = 0%) shows, as expected, the worst performance in terms of flexibility exploitation. The corresponding CDF is significantly shifted towards higher flexibility power values, indicating that larger control actions are required to compensate for the lack of monitoring information. This behavior is quantitatively confirmed by Table 4, where only 73.6% of the cases require a flexibility power lower than the benchmark reference value of 45.83 kW.

Introducing monitoring devices on 50% of the network nodes results in a systematic reduction in the required flexibility power. All SH = 50% scenarios exhibit a leftward shift in the CDF compared to the worst scenario, indicating that improved observability allows for more efficient and targeted control actions. The numerical results show that the percentage of cases with flexibility power below the benchmark increases to 78.3%, 79.1%, and 79.5% for ACCs equal to 2%, 1%, and 0.5%, respectively. While the differences among accuracy levels are limited, these results confirm that partial monitoring already enables a noticeable improvement in flexibility utilization.

When full monitoring coverage is considered (SH = 100%), the benefits become even more evident. In this case, the CDFs rapidly reach a cumulative probability of 100% at very low flexibility power values, indicating that the required control actions are consistently lower than the benchmark reference. This is confirmed by Table 4, where all SH = 100% scenarios achieve 100% of cases with flexibility power lower than the reference value, regardless of the adopted measurement accuracy. This outcome highlights that full observability allows the flexibility resources to be exploited in a highly efficient manner, minimizing unnecessary control effort.

Overall, the figure and the table demonstrate that increasing the monitoring penetration significantly reduces the amount of flexibility power required to operate the network securely, while the impact of measurement accuracy remains secondary.

The combined analysis of residual congestions, voltage violations, and flexibility power provides a comprehensive assessment of the impact of monitoring infrastructure characteristics on network operation.

## 7. Discussion

Across all performance indicators, the share of the distributed monitoring systems emerges as the dominant factor influencing system performance. This outcome is consistent with the fact that LV networks are typically characterized by sparse monitoring, where limited observability amplifies the impact of estimation errors and forces the controller to operate with incomplete system awareness. Increasing the share of monitored nodes consistently leads to fewer residual congestions, a near-complete elimination of voltage violations, and a substantial reduction in the required flexibility power. In particular, full monitoring coverage (SH = 100%) ensures optimal or near-optimal performance in all considered metrics, independently of the adopted measurement accuracy.

Measurement accuracy, while still beneficial, plays a secondary role compared to sensor penetration. Its impact becomes more visible in scenarios characterized by high observability, especially for congestion mitigation, whereas voltage regulation and flexibility activation already achieve satisfactory results even with moderate accuracy levels when sufficient monitoring coverage is available. Nevertheless, accuracy remains relevant in operational practice, especially when control actions are close to technical limits, where even small measurement deviations may translate into incorrect flexibility dispatch or delayed congestion detection.

The results also highlight different sensitivities among the analyzed indicators. This difference suggests that voltage regulation can tolerate partial visibility due to the local nature of voltage support, whereas congestion mitigation requires a more detailed representation of current flows and branch loading, and thus it is inherently more sensitive to measurement uncertainty and incomplete sensing. Voltage violations are the least critical issue and can be effectively mitigated with partial monitoring. Residual congestions exhibit a stronger dependence on both SH and ACC, requiring denser and more accurate monitoring infrastructures. Flexibility power lies between these two extremes, benefiting significantly from increased observability and enabling more efficient use of control resources as SH increases.

From a system planning and operation perspective, these findings suggest that investing in a widespread deployment of monitoring devices is a key enabler for effective congestion management, voltage control, and efficient flexibility utilization. In this respect, the monitoring infrastructure should be regarded as a foundational enabler of flexibility-based services, rather than a complementary asset, since the quality of the control outcome is strongly conditioned by the availability and reliability of measurement inputs. Once an adequate monitoring penetration is achieved, improvements in measurement accuracy can further refine performance, but their marginal benefit is lower compared to increasing sensor penetration.

Overall, the obtained results highlight that uncertainty related to the monitoring infrastructure is not a secondary aspect but rather a key limiting factor for the effectiveness of smart grid services in LV distribution networks. In particular, sensor penetration emerges as the dominant driver of performance, since a higher level of observability significantly improves both voltage regulation and congestion mitigation, while measurement accuracy mainly provides additional refinement once adequate monitoring coverage is ensured. This confirms that monitoring infrastructure characteristics should be treated as a primary planning variable for DSOs, as insufficient sensing capabilities may lead to conservative flexibility activation and reduce the reliability of control actions under unreliable operating conditions.

These findings indicate that the design of smart grid services cannot be decoupled from the deployment strategy of sensors and metering devices, because the achievable performance level is intrinsically bounded by the degree of network observability.

## 8. Conclusions

This paper investigates the impact of both the penetration and the measurement quality of distributed monitoring systems in LV grids on the management of smart grid services for both voltage regulation and congestion reduction. The proposed analysis demonstrates that the performance of modern voltage regulation and congestion management strategies is intrinsically linked to the technical characteristics of the underlying monitoring infrastructure. The share, representing the density of sensor deployment, emerged as the most influential parameter across all investigated scenarios. Higher sensor penetration consistently leads to the reduction in residual congestions, the near-total elimination of voltage violations, and more efficient utilization of flexibility resources. Specifically, when full monitoring coverage is achieved, the smart grid service framework ensures optimal performance regardless of the specific measurement accuracy adopted for monitoring devices.

The study further highlights that different network issues exhibit a different sensitivity with respect to the penetration and measurement quality of the distributed monitoring systems. Voltage stability was found to be more robust, achieving acceptable operational profiles even with moderate levels of sensor penetration. In fact, in the case of the absence of distributed monitoring systems (i.e., SH = 0%), the percentage of cases with fewer than one voltage violation is equal to 93.9%, achieving 98.9% with 50% coverage of monitoring systems (considering an accuracy of 0.5% in power measurement). A similar trend can be identified by looking at the required power flexibility needed for smart grid services. According to the obtained results, the percentage of cases where the required power flexibility is lower than the benchmark reference value achieves 100% with a full coverage of distributed monitoring systems, even when considering the worst measurement quality analyzed in this study (i.e., 2%). Within the smart grid service framework, the reduction in required power flexibility confirms that increased observability allows for more targeted and less conservative control actions.

Conversely, effective congestion management demonstrates a more critical dependence on both the number of monitored nodes and the measurement quality, requiring a denser and more accurate monitoring network to manage line overloads effectively. This was observed as the percentage of cases with fewer than one residual congestion is very low both in the case of the absence of monitoring infrastructure (i.e., 46.2%) and in the case of 50% monitoring system penetration in the LV grid (i.e., 55.3% in the case of a fixed accuracy of 0.5%).

Moreover, the smart grid service for congestion reduction exhibits a greater sensitivity to the measurement quality with respect to the smart grid service for voltage regulation. In fact, considering the full coverage of distributed monitoring systems (i.e., SH = 100%), the percentage of cases with fewer than one residual congestion increases from 91.9% to 97.9% when passing from 2% to 0.5% of accuracy in the power measurement. Nonetheless, the impact of the measurement quality remains secondary compared to the impact of expanding the monitoring penetration.

The outcomes of this analysis may be crucial for distribution system operators planning infrastructure upgrades who are interested in ensuring a secure, cost-effective transition toward active distribution grid management.

The main limitations of this work are linked to the assumption of a single accuracy class for all the monitoring systems deployed in each presented scenario and to the reduced number of uncertainty sources that was included in the analysis.

The development of a more complex assessment procedure of the impact on smart grid services that integrates other uncertainty sources (such as the uncertainty associated with the network parameters of the grid lines and with the placement of measurement devices in the network) could be a useful upgrade of the presented results.

In addition, future research activities should also consider the impact of user behavior, distributed generation variability, and potential cyber-physical threats like False Data Injection Attacks, testing the framework on different and real LV grids.

Finally, the technical analysis reported in this paper could be enhanced with an economic evaluation, considering the best trade-off between the number and the quality of the distributed monitoring systems deployed by distribution system operators and the costs allocated to flexibility request activation and network issue resolution.

## Figures and Tables

**Table 1:** Definition of simulation scenarios based on distributed monitoring system share and measurement accuracy.

	Scenarios						
**SH [%]**	0	50	50	50	100	100	100
**ACC [%]**	0	2	1	0.5	2	1	0.5

**Table 2:** Percentage of cases with fewer than one residual congestion.

	Scenarios						
**SH [%]**	0	50	50	50	100	100	100
**ACC [%]**	0	2	1	0.5	2	1	0.5
**CDF [%]**	46.2	53.5	54.3	55.3	91.9	97.2	97.9

**Table 3:** Percentage of cases with fewer than one voltage violation for each scenario.

	Scenarios						
**SH [%]**	0	50	50	50	100	100	100
**ACC [%]**	0	2	1	0.5	2	1	0.5
**CDF [%]**	93.9	98.6	98.8	98.9	100.0	100.0	100.0

**Table 4:** Percentage of cases where the required flexibility power is lower than the benchmark reference value.

	Scenarios						
**SH [%]**	0	50	50	50	100	100	100
**ACC [%]**	0	2	1	0.5	2	1	0.5
**CDF [%]**	73.6	78.3	79.1	79.5	100.0	100.0	100.0

## Data Availability

The data presented in this study are available on request from the corresponding author.
